# Children hospitalized with community-acquired pneumonia complicated by effusion: a single-centre retrospective cohort study

**DOI:** 10.1186/s12887-023-04004-2

**Published:** 2023-04-19

**Authors:** Gelila Alemayheu, Claire S. J. Lee, Laura K. Erdman, Jacqueline Wong, Candy Rutherford, Marek Smieja, Sarah Khan, Jeffrey M. Pernica

**Affiliations:** 1 School of Medicine, Western University, London, ON Canada; 2 Faculty of Medicine, University of Toronto, Toronto, ON Canada; 3 Department of Pediatrics, Faculty of Health Sciences, McMaster University, 1280 Main St West, L8S 4K1 Hamilton, ON Canada; 4 Hamilton Regional Laboratory Medicine Program, Hamilton, ON Canada; 5 Department of Pathology and Molecular Medicine, Faculty of Health Sciences, McMaster University, Hamilton, ON Canada; 6 Department of Health Research Methods, Evidence, and Impact, Faculty of Health Sciences, McMaster University, Hamilton, Canada

**Keywords:** Pneumonia, Pleural effusion, Epidemiology, Procedural drainage, Microbiology

## Abstract

**Objectives:**

To describe children hospitalized with community-acquired pneumonia complicated by effusion (cCAP).

**Design:**

Retrospective cohort study.

**Setting:**

A Canadian children’s hospital.

**Participants:**

Children without significant medical comorbidities aged < 18 years admitted from January 2015-December 2019 to either the Paediatric Medicine or Paediatric General Surgery services with any pneumonia discharge code who were documented to have an effusion/empyaema using ultrasound.

**Outcome measures:**

Length of stay; admission to the paediatric intensive care unit; microbiologic diagnosis; antibiotic use.

**Results:**

There were 109 children without significant medical comorbidities hospitalized for confirmed cCAP during the study period. Their median length of stay was 9 days (Q1-Q3 6–11 days) and 35/109 (32%) were admitted to the paediatric intensive care unit. Most (89/109, 74%) underwent procedural drainage. Length of stay was not associated with effusion size but was associated with time to drainage (0.60 days longer stay per day delay in drainage, 95%CI 0.19-1.0 days). Microbiologic diagnosis was more often made via molecular testing of pleural fluids (43/59, 73%) than via blood culture (12/109, 11%); the main aetiologic pathogens were *S. pneumoniae* (40/109, 37%), *S. pyogenes* (15/109, 14%), and *S. aureus* (7/109, 6%). Discharge on a narrow spectrum antibiotic (i.e. amoxicillin) was much more common when the cCAP pathogen was identified as compared to when it was not (68% vs. 24%, p < 0.001).

**Conclusions:**

Children with cCAP were commonly hospitalized for prolonged periods. Prompt procedural drainage was associated with shorter hospital stays. Pleural fluid testing often facilitated microbiologic diagnosis, which itself was associated with more appropriate antibiotic therapy.

## Background

Community-acquired pneumonia (CAP) remains one of the most common reasons for paediatric hospitalization in North America [1], despite the fact that recent prospective randomized trials have shown that hospitalization occurs rarely (0–6%) with paediatric CAP managed in the outpatient setting with oral antimicrobials [2–4]. An even smaller proportion of children with CAP go on to develop pleural effusion or empyaema, though paediatric hospitalists regularly encounter these cases. In the pre-pneumococcal conjugate vaccine era, one retrospective cohort study done at 8 Canadian children’s hospitals over a 3-year period identified 251 children with effusion/empyaema [5]. A decrease in pneumococcal disease was widely observed after the introduction of the seven-valent pneumococcal vaccine (PCV7)[6]; paradoxically, some observed an increase in children with CAP complicated by effusion/empyaema (cCAP) during this time period [7–9], which may have been provoked by the H1N1 influenza pandemic [10]. Later, the 13-valent pneumococcal vaccine (PCV13) was introduced; this was followed by a decrease in paediatric cCAP in some jurisdictions [11, 12], though not in others [13].

The microbiologic aetiology of paediatric cCAP has been previously investigated; in high-income countries, the most common causative pathogen has been consistently shown to be *Streptococcus pneumoniae* [13–15], though this is not a universal finding [16]. Other common causes of paediatric cCAP include *Streptococcus pyogenes* (group A streptococcus), *Staphylococcus aureus*, and other streptococci [14, 17–19]. Microbiologic diagnosis is important because it facilitates the provision of optimal antibiotic therapy; penicillin (or amoxicillin/ampicillin) is the most appropriate treatment for most of these pathogens, excepting the majority of *S. aureus* isolates. While traditional bacterial culture of blood and/or pleural fluid is rarely positive, identification of the infecting pathogen can be greatly facilitated using molecular diagnostics on pleural fluid [14, 19].

Management of children with cCAP always includes antibiotic treatment, but the need for drainage and/or debridement can vary, depending on the size and nature of the effusion/empyaema [17, 18, 20]. It is thought that effusions begin as free-flowing fluid that then can develop loculations and further progress to a grossly purulent liquid that can evolve into a fibrinous peel [21]. Generally speaking, small effusions rarely require drainage, whereas those that are large (such that they contribute to respiratory symptoms through mechanical or other means) or that are present in critically ill children require consideration of evacuation to expedite clinical improvement [17, 18]. Unfortunately, chest radiographs may not reliably distinguish between effusion and consolidation, and ultrasound performance is operator-dependent [21]; consequently, for those without very small or very large effusions, whether procedural drainage offers more potential benefit than harm is often unclear at the outset of hospitalization. Guidelines published a decade ago by the Infectious Disease Society of America/Pediatric Infectious Disease Society [17] and by the American Pediatric Surgical Association [21] noted that outcomes have been found to be similar in children (without advanced-stage empyaema) treated with chest thoracostomy tube placement with intrapleural instillation of fibrinolytic agents as compared to those treated with video-assisted thoracoscopic surgery (VATS); this formed the basis for their suggestion that either thoracostomy with fibrinolysis or VATS can be used depending on local expertise or the particulars of the patient, with the recognition that thoracostomy plus fibrinolysis is generally a simpler first step.

In summary, paediatric cCAP is a pathologic entity that is commonly encountered by paediatricians, infectious disease physicians, and surgeons, whose epidemiology and microbiology has varied (and will likely vary in coming years), for which optimal management strategies have not been definitively identified. The purpose of this study was to describe a cohort of children with cCAP managed at a Canadian children’s hospital.

## Methods

### Study design and setting

This was a retrospective, single-centre, cohort study. McMaster Children’s Hospital (MCH) is a tertiary care centre (serving a catchment of approximately 2 million people) that had 159 beds and admitted approximately 6500 children/year during the study period. The standard of care for children hospitalized with cCAP included culture of both blood and pleural fluid using traditional microbiologic methods (blood is cultured using the BacT/ALERT [bioMérieux, France] automated system). A lab-developed Taqman-based multiplex PCR assay (targets *S. aureus, S. pneumoniae, S. pyogenes, S. anginosus*) was also often utilized for the testing of pleural fluids if pathogens were not detected using culture-based methods. At MCH, for all children with chest tubes placed for cCAP, it is standard of care to instill three doses (q24h) of tissue plasminogen activator into the pleural space, starting the day of thoracostomy. The province of Ontario, where the study was conducted, incorporated PCV13 into its universal vaccine schedule beginning in 2010. This study was approved by the Hamilton Integrated Research Ethics Board (2020–8336). The necessity for informed consent was waived for this chart review.

### Study population

Children less than 18 years of age admitted to the Paediatric Medicine or Paediatric General Surgery services between 1 January 2015-31 December 2019 with any discharge diagnosis of any pneumonia (A403, B593, B960, B961, J100, J110, J120, J122, J123, J128, J129, J13, J14, J150, J151, 152, J153, J154, J155, J156, J157, J158, J159, J168, J170, J171, J172, J173, J180, J181, J182, J189, J851, J852) were eligible for inclusion. Children were then excluded if they did not have complicated pneumonia, which was defined as having a drainable effusion seen in their last ultrasound report before drainage or before discharge (if no drainage procedure was done). Children were also excluded if they had any of the following: cystic fibrosis, chronic lung disease, tracheostomy, congenital heart disease, history of repeated aspiration or velopharyngeal incompetence, malignancy, conditions requiring treatment with immune suppressants, primary immunodeficiency, advanced HIV infection, chronic renal dysfunction, chronic hepatic dysfunction, history of active tuberculosis within the past year, suspected active tuberculosis, or lung abscess diagnosed within the past six months.

### Study outcomes

Data collection was performed using a standardized web-based data collection form (Research Electronic Data Capture, REDCap) by two investigators (GA, CL). Baseline covariates of interest were: age, gender, antibiotic treatment before admission, chest radiograph findings, ultrasound findings, drainage date, and microbiologic testing results (nasopharyngeal swabs, blood culture, pleural fluid culture, and pleural fluid multiplex PCR testing). Pneumococcal vaccination status was not abstracted because this information was not documented reliably within the medical record; in Ontario, vaccination records are not readily accessible to clinical care providers. During the study period, in the province, pneumococcal vaccination coverage varied from 74.1 to 79.0% in 7 year-olds [22]. The primary outcome was length of stay (LOS); other outcomes of interest were admission to the paediatric intensive care unit (PICU) and antibiotic treatment during hospitalization. At MCH, not all children with cCAP are cared for in the PICU; typical indications for PICU admission for this patient population include the need for ventilatory support (e.g. high-flow oxygen, continuous positive airway pressure, bilevel positive airway pressure, mechanical ventilation) and haemodynamic support (e.g. crystalloid boluses, vasopressor infusions).

### Statistical analysis

Descriptive statistics to describe baseline characteristics were reported as count (percent) for categorical variables, and mean (standard deviation) or median (first quartile-third quartile, labeled as interquartile range [IQR]) for continuous variables depending on the distribution. Given that LOS varied considerably between participants, antibiotic treatment durations were also presented as proportions (i.e. duration of antibiotic treatment divided by LOS). Qualitative findings (i.e. small/small to moderate/moderate/moderate to large/large) documented in ultrasound reports from the attending radiologist were abstracted. T-tests or linear regression were used to compare normally-distributed continuous variables. Poisson regression was used to compare count variables. Chi-square or Fisher exact testing was used to compare categorical variables between groups. Alpha was set at 0.05, with no adjustments for multiple comparisons in this exploratory study. No imputation of missing data was done. Analyses were conducted using Stata v11.2 (College Station, TX).

### Patient and public involvement

The public was not involved in the design or reporting of the study.

## Results

There were a total of 2111 children admitted to the Paediatric Medicine or Paediatric General Surgery services with a pneumonia diagnosis code during the study period. Of those, only 109 were eligible (i.e. had effusion/empyaema confirmed by imaging and did not have pre-existing comorbidities). As shown in Table 1, the median age of the study subjects was 4.7 years and there was an even biologic sex split. A majority (78%) had received some antibiotic therapy prior to admission to MCH.

**Table 1:** Demographic characteristics

Covariate	
Median age (Q1-Q3)	4.7 years (3.2–6.6 years)
Biologic sex	
Female Male	56 (51%) 53 (49%)
Year of admission	
2015 2016 2017 2018 2019	23 23 25 16 22
Had nasopharyngeal viral testing	88 (80%)
Rhino/enterovirus RSV Influenza hMPV Parainfluenza *Mycoplasma* Adenovirus At least one detectable pathogen Two pathogens detected Negative for all pathogens assayed	22 (25%) 4 (5%) 4 (5%) 2 (2%) 2 (2%) 2 (2%) 1 (1%) 34 (39%) 3 (3%) 54 (61%)
Receipt of antibiotics before admission^1^	
None Amoxicillin Ceftriaxone Vancomycin Ampicillin Clarithromycin or azithromycin Amoxicillin/clavulanate Cefprozil or cefuroxime	24 (22%) 23 (21%) 59 (54%) 26 (24%) 9 (8%) 22 (20%) 8 (7%) 9 (8%)

1. Categories are not mutually exclusive

The median LOS was 9 days and 15 children (14%) were admitted for two weeks or more. There were 35/109 (32%) who were admitted to the PICU; the median PICU LOS for these subjects was 5 days (Q1-Q3 2–8 days). The median duration of ceftriaxone treatment received after admission was 6 days (Q1-Q3 3–9 days); more subjects received > = 2 weeks of treatment (6/109, 6%) than did not receive it at all (4/109, 4%). Subjects received ceftriaxone after admission for a median of 83% of their days in hospital (Q1-Q3 40-100%). Only 34/109 (31%) received vancomycin after admission; for those who were prescribed vancomycin, the median duration of vancomycin treatment received was 2 days (Q1-Q3 1–4 days), corresponding to a median of 25% of their days in hospital (Q1-Q3 13–44%). There were 28/109 (26%) who received ampicillin after admission; for those who were prescribed ampicillin, the median duration received was 4.5 days (Q1-Q3 3–7 day), with subjects receiving ampicillin for a median of 50% of their days in hospital (Q1-Q3 39–65%).

A majority (81/109, 74%) of study subjects had procedural drainage completed. Drainage was performed by Interventional Radiology (69%), General Surgery (17%), Emergency Medicine/Critical Care Medicine (9%), or was not specified (5%). There were only 7/81 (9%) who went on to have a thoracotomy by the General Surgery service. Of those who were drained, there were 77 (95%) that had qualitative assessment of the size of the effusion by the attending radiologist. Few subjects with a small effusion underwent drainage (2/12, 17%). Significantly more children with non-small effusions were drained (5/7 with small-moderate, 71%; 27/30 with moderate, 90%; 7/8 with moderate-large, 88%; 19/20 with large, 95%; p < 0.001, Fisher’s exact test).

There were 12/109 (11%) with positive blood cultures (10 *S. pneumoniae*, 1 methicillin-sensitive *S. aureus* (MSSA), and 1 *S. pyogenes*). There were fewer (10/81, 12%) positive pleural fluid cultures (7 *S. pyogenes*, 2 MSSA, and 1 *S. anginosus*). Molecular testing of pleural fluids had a much higher yield, with 43/59 (73%) positive; there were 30/43 (70%) positive for *S. pneumoniae*, 8/43 (19%) positive for *S. pyogenes*, and 5/43 (12%) positive for MSSA. Overall, taking all microbiologic testing into account, the microbiologic cause of the subjects’ illnesses was as follows: *S. pneumoniae* (40/109, 37%), S. pyogenes (15/109, 14%), methicillin-sensitive *S. aureus* (7/109, 6%), and *S. anginosus* (1/109, 1%). No cases of proven methicillin-resistant *S. aureus* (MRSA) were identified, and 46 cases (42%) did not have a definitive aetiology found. There were no discordant microbiologic results.

There appeared to be differences in the in-hospital antibiotic treatment of those who had a microbiologic cause identified as compared to those who did not. Those children with the cause of their cCAP identified received 24% less ceftriaxone (59% of total LOS vs. 83% of total LOS, p < 0.0001 by t-test) and 15% more ampicillin (19% of total LOS vs. 4% of total LOS, p = 0.002 by t-test); the duration of vancomycin was not found to be different between groups. At discharge, 54 (49%) of subjects received amoxicillin; this varied from 39-43% in 2015–2017 to 62–68% in 2018–2019. Those with a microbiologic diagnosis received amoxicillin much more often as compared to those who did not (43/63 [68%] vs. 11/46 [24%], p < 0.001 by Fisher’s exact test). There were 33 subjects (30%) who were discharged on amoxicillin/clavulanate; this agent was given much less frequently to those with a microbiologic diagnosis as compared to those who did not (10% vs. 90%, p < 0.001 by Fisher’s exact test).

The median time to drainage was 1 day after admission (Q1-Q3 1–3 days after admission); 4 subjects (5% of those drained) had the procedure completed 7 days or more after admission. The time to drainage did not appear to differ between subjects with different effusion sizes either using visual inspection or by Poisson regression. LOS was shorter for subjects with small effusions than those with effusions that were larger than ‘small’ (difference 3.6 days, 95%CI 0.55–6.6 days, p = 0.021). However, when only subjects that underwent drainage were included, LOS was not found to be longer in those with larger effusions. For those undergoing drainage, LOS was found to be positively associated with the time to drainage (0.60 days longer stay per day delay in drainage, 95%CI 0.19-1.0 days, p = 0.004).

## Discussion

This single-centre retrospective cohort study found that young children were often hospitalized for prolonged periods at a Canadian children’s hospital because of CAP complicated by effusion/empyaema in 2015–2019. Subject LOS in-hospital was also found to be positively associated with time until procedural drainage. Finally, broad-spectrum antibiotic use was common, especially in those who did not have the aetiology of their pneumonia definitively demonstrated.

Thankfully, mortality associated with paediatric CAP is very rare [23]. Having said that, we found that morbidity associated with cCAP was substantial, even in children without any significant medical comorbidities. Our study subjects both required much longer hospitalizations and were more frequently admitted to the PICU as compared to children admitted to 3 American hospitals because of (predominantly) uncomplicated CAP [23]. We note that the median LOS of our study subjects was similar to that of children with empyaema hospitalized two decades ago at 8 Canadian children’s hospitals [5], underscoring the importance of urgently improving the prevention or management of children with cCAP.

There is uncertainty regarding the need for procedural intervention in the many children with cCAP who do not have very large or very small effusions and are neither very ill nor very well [17, 18, 20]. Anecdotally, we have observed different clinicians express diverse viewpoints about whether a given patient with cCAP would benefit from drainage, often resulting in a decision to ‘closely observe’. The idea that clinicians are carefully seeking the ‘right’ answer for each individual is laudable in nature; however, the ’wait-and-see’ approach also has potential harm, as we observed that delays in drainage of parapneumonic collections were associated with prolongations of hospital stay. In this type of study, however, we cannot prove causality, and so cannot say with certainty that there were not other factors associated with delays in drainage that themselves caused a longer LOS. We would note that for many other infection syndromes associated with the development of suppurative collections, source control is considered to be important to expedite recovery and optimize eventual outcome.

We found that pleural fluid sampling (and subsequent molecular testing) was associated with much higher rates of microbiologic diagnosis than traditional culture-based techniques, in keeping with previous studies [19]. One might expect that microbiologic diagnosis would facilitate antimicrobial selection, and our results would lend support to this hypothesis, as study subjects with an identified pathogen were much more likely to have been transitioned from broad-spectrum to narrow-spectrum antibiotics. We would suggest that optimization of antimicrobial therapy constitutes a substantial benefit not only to the individual patient (who is much less likely to experience direct side effects) but also to the hospital and greater community as well, since the widespread use of broad-spectrum antibiotics will stimulate the development of antimicrobial-resistant organisms.

This study was performed prior to the COVID-19 pandemic, which completely changed the typical seasonality of respiratory viruses in many jurisdictions [24, 25]. Given the known interplay between respiratory viruses and bacterial pulmonary disease [26, 27], it is not surprising that differences in the incidence of invasive pneumococcal disease and bacterial pulmonary disease were also observed during periods of non-pharmaceutical interventions to prevent SARS-CoV-2 transmission, when respiratory virus positivity rates were at historic lows [28, 29]. Consequently, it may be that the aetiology and prognosis of children hospitalized with cCAP at our centre in the future may differ from our past experience. For example, in late 2022, we (anecdotally) observed uncharacteristically high RSV hospitalization rates at our centre, followed by (apparently) substantially higher rates of invasive group A streptococcal disease and cCAP. Others have also noted changes in the microbiologic aetiology of cCAP in the post-pandemic era [30]. At this stage, it is difficult to accurately predict respiratory viral circulation patterns [31] or the future incidence or epidemiology of paediatric cCAP.

The main limitation of this study is that it followed a cohort retrospectively at only a single site. It may well be that the children described, and the practices of the physicians that cared for them, are not representative of other sites in Canada and elsewhere. In this chart review, pneumococcal vaccine status could not be confirmed; consequently, we were not able to explore whether this significantly influenced cCAP aetiology at our centre. There have also been other studies demonstrating that cCAP microbiology is different in different centres and regions [32]. A prospective study would give more insight into causal relationships and a multicentre study would increase generalizability of the findings.

## Conclusion

At our centre, in the PCV13 era, children were commonly hospitalized for prolonged periods because of cCAP. Prompt drainage of effusions was found to be associated with shorter hospital stays and facilitated the identification of the aetiology of the infection. Microbiologic diagnosis was associated with more appropriate narrower-spectrum antibiotic treatment.

## Data Availability

The datasets used in this study are available from the corresponding author on reasonable request.
